# Inflammation-related research within the field of bladder cancer: a bibliometric analysis

**DOI:** 10.3389/fonc.2023.1126897

**Published:** 2023-06-07

**Authors:** Zhixuan Deng, Ning Tang, Wanyan Xiong, Xu Lei, Tengfei Zhang, Ning Yang

**Affiliations:** ^1^Department of Urology, The Second Affiliated Hospital, Hengyang Medical School, University of South China, Hengyang, Hunan, China; ^2^Department of Orthopaedics, Third Xiangya Hospital of Central South University, Changsha, Hunan, China; ^3^Department of Respiratory Medicine, The Affiliated Nanhua Hospital, Hengyang Medical School, University of South China, Hengyang, Hunan, China

**Keywords:** bladder cancer, inflammation, bibliometrics, CiteSpace, VOSviewer

## Abstract

**Background:**

In recent years, the link between inflammation and bladder cancer(BC) has received much attention. However, there were no relevant bibliometric studies to analyze the inflammation-related research within this field of BC.

**Methods:**

We selected Web of Science Core Collection (WOSCC) as the data source to obtain articles and reviews on inflammation-related research within te field of BC from WOSCC’s inception to October 10, 2022. The collected data were meticulously and manually screened, after which we used VOSviewer, CiteSpace, Biblioshiny and an online analysis platform (https://bibliometric.com/) to perform bibliometric analysis on the data and visualize the results.

**Results:**

A total of 4301 papers related to inflammation-related research within this field of BC were included in this study.The number of publications has steadily increased over the last decades (R²=0.9021). The top contributing country was the United States, O’Donnell, Michael A was the most published authors, the leading contributing institution was the University of Texas, and the leading contributing journal was JOURNAL OF UROLOGY. The keywords co-occurrence analysis indicated that “immunotherapy,” “inflammation-related biomarkers,” and “tumor microenvironment” were the hot spots and frontiers of research in this field.

**Conclusion:**

This study clarifies the contribution of countries, institutions, authors, and journals in inflammation-related research within this field of BC through a bibliometric approach and identifies research hotspots and frontiers in the field. Notably, these findings can help researchers to understand more clearly the relationship between inflammation and BC.

## Introduction

Bladder cancer (BC) is a malignancy with a high incidence that usually presents as painless, intermittent hematuria visible to the naked eye (1). The Global Cancer Statistics 2020 report shows approximately 573,000 new cases and 213,000 deaths worldwide (2).Notably, the development and progression of BC are associated with many factors, including smoking, alcohol abuse, and exposure to polycyclic aromatic hydrocarbons or aromatic amines (3, 4). More recently, a close association between inflammation and the development of BC has also been found (5).

Inflammation is a defense response of the organism to stimuli, a mechanism by which the immune system neutralizes or eliminates injurious stimuli and initiates regeneration or healing (6). During the development of BC, excessive or persistent inflammation has been shown to promote BC development by activating a range of inflammatory molecules and signals within the tumor microenvironment (7, 8).Consequently, the relationship between inflammation and BC is gaining more scholarly attention (9–11). For instance, XH’s study revealed the role of UBC9 in regulating inflammatory signaling in BC and that lack of UBC9 leads to BC progression, mainly through inflammatory activation and stem cell-like population formation (12). A clinical study by WZ and his colleagues demonstrated that the systemic immune-inflammation index (SII) was an inflammation-related biomarker with more predictive power than traditional biomarkers and could predict the prognosis of patients undergoing radical cystectomy (RC) prognosis (13). Therefore, these indicate that inflammation is closely related to BC’s occurrence, development, and prognosis.

Faced with vast and rapidly developing information in this field, researchers must devote a lot of time and effort to searching and reviewing publications. As a result, we need new approaches to extract and analyze the literature to obtain necessary and relevant information. Bibliometrics is a well-recognized method for the systematic evaluation of a research field, which can clarify the contributions of various countries and institutions in a field, identify active researchers and potential collaborators, and describe trends in the evolution of research directions (14, 15). Many bibliometric articles on BC or inflammation have recently been published (16, 17). Notably, “inflammation” is one of the most critical topics in a bibliometric analysis of the top 100 most cited manuscripts in BC research published in 2020 (16). However, no investigator has published a bibliometric study that includes both BC and inflammation. To fill this research gap, we provided an accurate description of the literature in inflammation-related research within this field of BC through bibliometric approaches.

## Methods

### Data source

The Web of Science(WOS) database is the data source for this study. In the academic world, WOS is considered one of the most widely literature databases and is widely used in bibliometric studies (18–20). Therefore, we selected the Web of Science Core Collection (WOSCC) of Science Citation Index Expanded (SCIE) to search publications on inflammation and BC.

### Data search strategy

**Figure 1:**
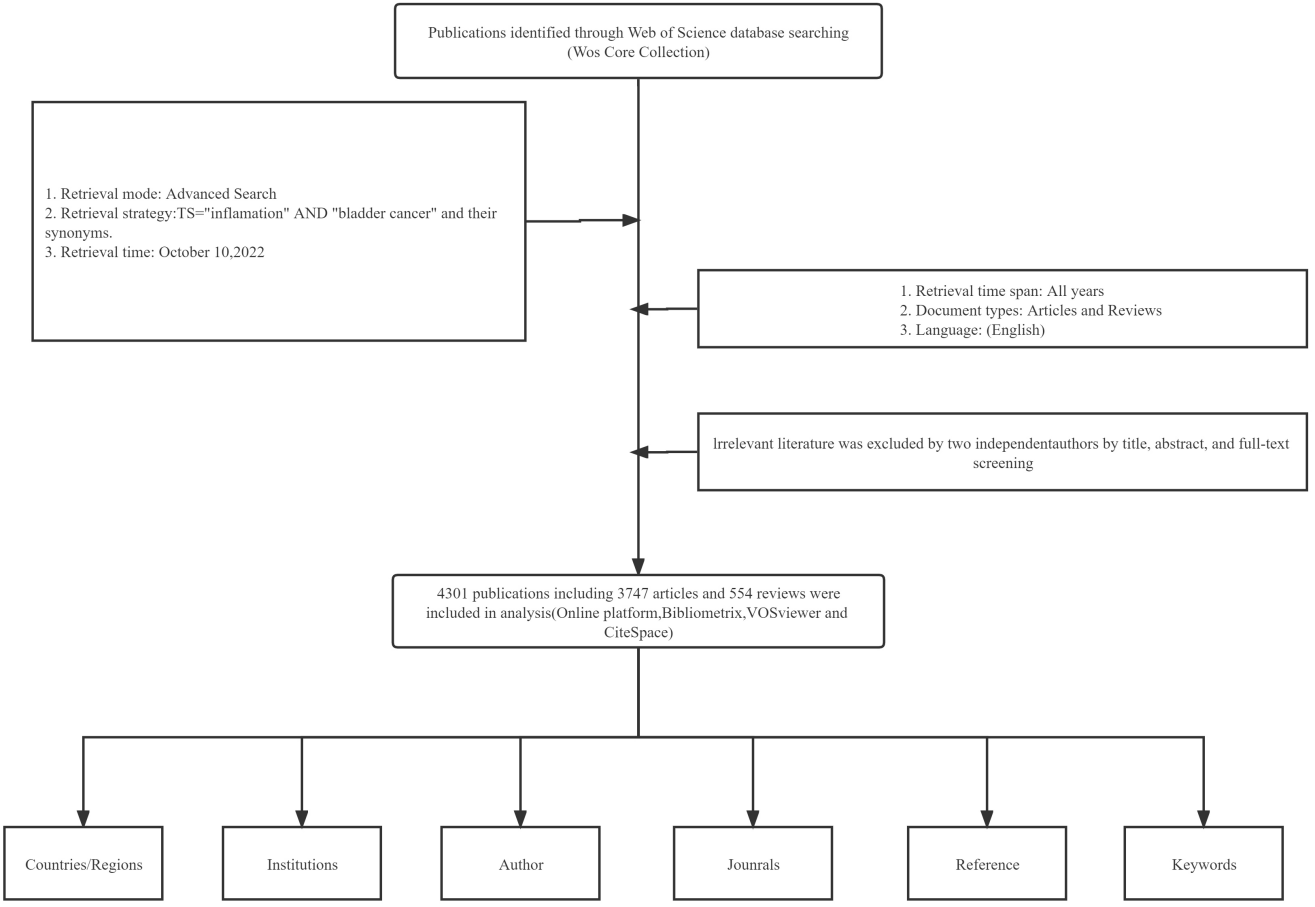
Flowchart of literature search and selection process. Two investigators manually reviewed the titles, abstracts, and even the full text of the literature to exclude relevant literature unrelated to the study topic.

Two authors (DZX and XWY) conducted a comprehensive literature search on October 10, 2022, for topics related to both “BC” and “inflammation”, TS = “inflammation” and “bladder cancer” and their synonyms (**Supplementary Table 1**) (16, 21, 22). The time frame was from the time of the WOS database to the present. Only papers published in English were included in our database. We only included articles and reviews in our research; other forms of publication, such as letters and meetings, were excluded. Two investigators (DZX and XWY) manually reviewed the titles, keywords, abstracts, and even the full text to exclude relevant literature unrelated to the study topic. The specific process is shown in **Figure 1**.

### Data extraction and collection

We downloaded the “complete record” (including author, source, title, publication, abstract, keywords, address, references, grants, etc.) of all the retrieved documents (4301 publications, including 3747 articles and 554 reviews) from the WOS database. Then we exported them in plain text and tab-delimited (win, UTF8) formats and named them “Downloaded_xx” for the bibliometric tool. The obtained bibliographic information was edited and filtered using Endnote 2020, followed by Microsoft Excel 2022 for basic analysis and graphing. It is worth mentioning that some inherent flaws within the WOS database were corrected. For example, publications from Taiwan were included in China, and England, Scotland, and Ireland were merged into the UK.

### Bibliometric analysis

We used the latest versions of bibliometric tools, including VOSviewer 1.6.18, CiteSpace 6.1.R3, and Biblioshiny 4.0, to obtain the most comprehensive data analysis results possible.

VOSviewer is a common bibliometric tool used to build and visualize bibliometric networks. We constructed a co-linear network graph of institutions, journals, and authors using various functions of VOSviewer. We also constructed a network and clustering graphs of authors’ keyword contributions based on text data. Finally, we processed the data that VOSviewer could not recognize, such as merging the author information “O’Donnell, Michael” with “O’Donnell, Michael A” and the keyword information “biomarkers” with “biomarker” and “bacillus Calmette-Guerin” with “BCG”.

CiteSpace has the advantage of being able to better reflect bibliometric data over time (23). Therefore, we used CiteSpace to visualize inter-country and inter-institutional collaborations, perform cluster analysis of keywords, and construct a dual-map overlay of journals. We also identified top 50 references with the strongest citation Bursts in the last 10 years through CiteSpace. Furthermore, we adjusted the parameter settings of CiteSpace as follows: selection criteria (g-index k=25), and pruning (Pathfinder, Pruning sliced networks),time span (1979–2022), and years per slice.

The online bibliometric platform Biblioshiny 4.0 in the R4.1.2 language environment was used to analyze the literature regarding the institution of attribution and author and subject terms. In addition, we constructed collaborative network graphs between countries using the online platform (https://bibliometric.com/).

## Results

### Publication outputs and trends

**Figure 2:**
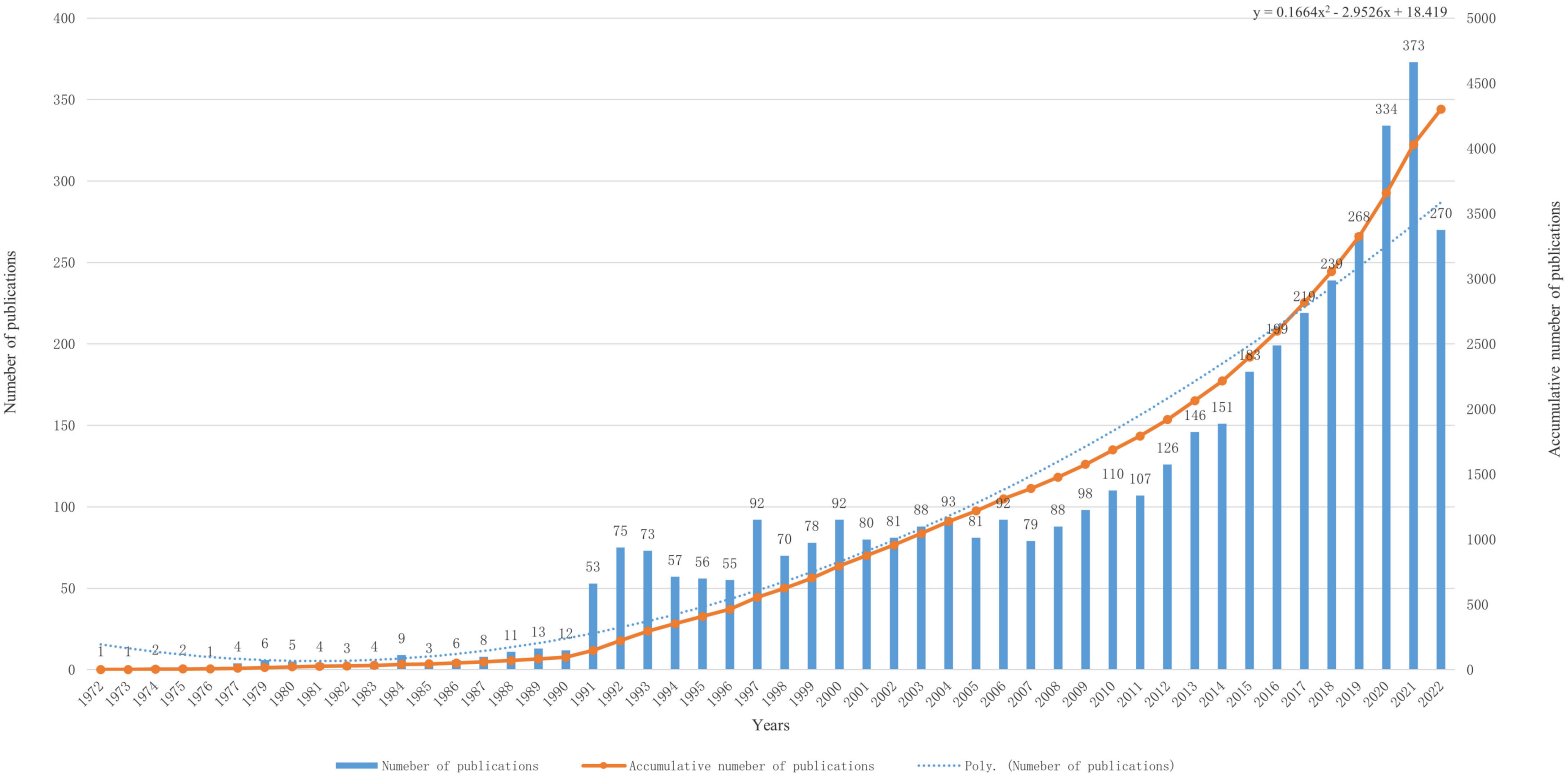
The specific amount of annual publications regarding BC and inflammation from 1972 to 2022. There is an upward trend in the number of articles on inflammation-related research within the field of bladder cancer from 1972 to 2022.The * sign in the figure indicates that the information on published publications in 2022 is incomplete because this study only collected relevant publications from 01/01/2022 to 10/10/2022.

We included 3747 articles (87.1%) and 554 reviews (12.9%) for the study’s total of 4301 publications. The first literature on inflammation and BC was published in 1972, and the number of publications skyrocketed from 1991, reaching a peak in 2019 (**Figure 2**). A fitted curve was made for the trend in annual publication volume, with the functional equation y = 0.1664x2 - 2.9526x + 18.419(R² = 0.9021, x is the year, y is the number of annual publications). Notably, this formula is a good reflection of the relationship between the year and the number of publications,however the final prediction may be subject to a slight error because only publications from 01/01/2022 to 10/10/2022 were included in this study.

### Most productive countries/regions

**Figure 3:**
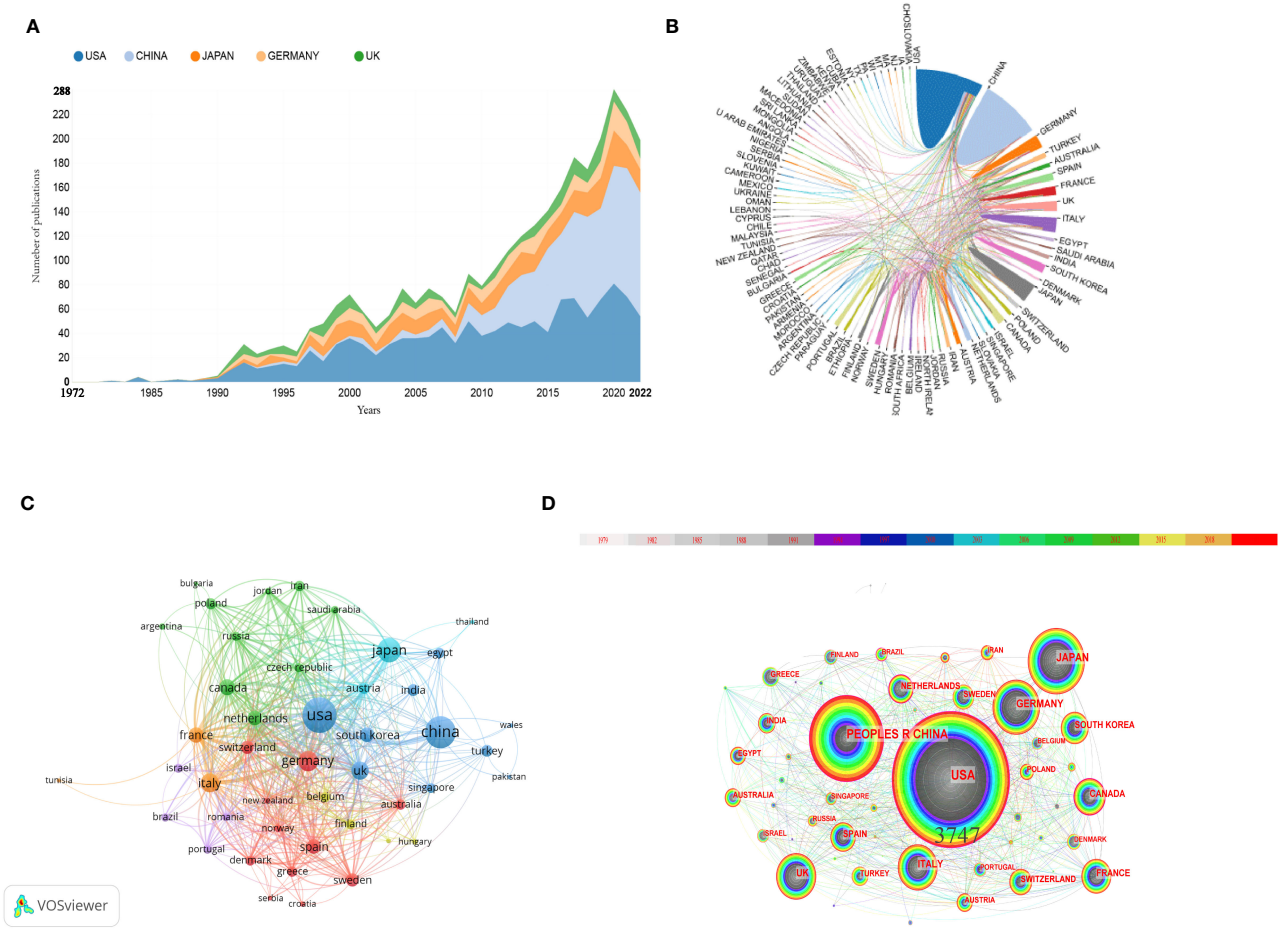
Map of the country’s contribution. **(A)** The annual publications of the top 5 countries from 1972 to 2022. **(B)** The international cooperation analysis among different countries. Thicker lines indicate closer cooperation between countries, while conversely, thinner lines indicate less close cooperation between countries. **(C)** The overlay visualization map of country co-authorship analysis conducted by VOSviewer. Each node represents a country, and the larger size of the node represents the larger number of publications in that country. The density of links between two nodes reflects the co-authorship relationship between countries. **(D)** The co-occurrence map of countries/regions in inflammation-related research within the field of bladder cancer (T≥25). Note: The node’s size reflects the frequency of co-occurrence, and a larger node indicates a higher frequency of contribution. Connected lines indicate co-occurrence relationships. The colors of nodes and lines represent different years, from 1972 to 2022, changing from gray to red over time. Nodes with purple circles imply higher intermediate centrality (>0.1).

In this study, we found that 92 countries/regions contributed to inflammation-related research within the field of BC. **Table 1** shows that the most published country was the United States of America(USA) (n=1290, 29.99%), followed by China and Japan. Among the top 5 countries in terms of the number of publications, each country’s number increased yearly (**Figure 3A**). The average number of citations of the documents from the USA was the highest (average citations per document=50.06) and higher than other countries/regions. As shown in **Figure 3B**, there is close collaboration between countries/regions in inflammation-related research within the field of BC. As shown in **Figure 3C**, the USA is at the core of global collaboration in inflammation-related research within the field of BC. Additionally.As shown in **Figure 3D**, China and the United States have continuously published papers in this field from 1972 to 2022, and the United States has published more papers than China before 1990.Seven countries were found to have a centrality ≥0.01 in **Figure 3D**.Furthermore, depending on the color of the nodes, we can see that the SWEDEN(1972)the first countries to conduct research in the field of inflammation and BC.

**Table 1:** Top 20 most productive countries in inflammation-related research within the field of bladder cancer.

Rank	Country/region	Publications, n	% of 4301	Citations	Average citations per document
1	USA	1290	29.99%	64577	50.06
2	CHINA	998	23.20%	20787	20.83
3	JAPAN	477	11.10%	14571	30.58
4	GERMANY	295	6.86%	11444	38.79
5	UK	260	6.04%	11273	43.22
6	ITALY	227	5.27%	8723	38.43
7	CANADA	161	3.74%	5894	36.61
9	FRANCE	155	3.60%	6655	42.94
10	NETHERLANDS	129	2.99%	4873	37.78
11	SOUTH KOREA	126	2.92%	3539	28.09
12	SPAIN	123	2.84%	5243	42.63
13	SWEDEN	102	2.37%	4082	40.02
14	SWITZERLAND	94	2.18%	3718	39.55
15	TURKEY	77	1.79%	1076	13.97
16	INDIA	74	1.72%	1331	17.99
17	AUSTRIA	69	1.60%	1951	28.28
18	POLAND	61	1.42%	912	14.95
19	GREECE	58	1.41%	1824	31.45
20	AUSTRALIA	54	1.26%	2016	37.33

The table is sorted by the number of publications. The number of citations was only counted for 4301 publications from the included local databases. Percentages are baed off of the number of publications (4301).

### Analysis of institutional output

**Figure 4:**
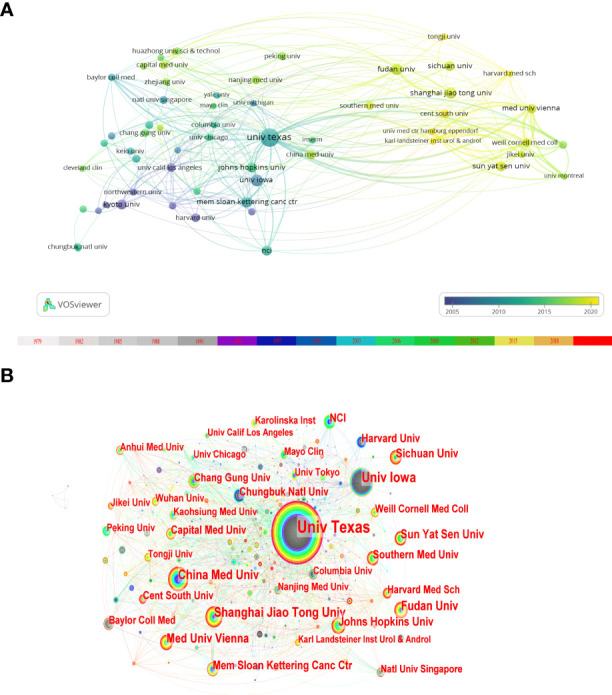
Map of the institutions’ contribution. **(A)** An overlay visualization map of institutional collaboration analysis performed by VOSviewer. Each institution is represented as a node. Links between two nodes indicate co-authorship. According to the color gradient in the lower right corner, different nodes are given different colors (based on larger average years of appearance (AAY). **(B)** Network visualization map of institutional collaborations generated by CiteSpace. In this map, a node represents an institution, and the size of each node represents its relative quantity of research output. Each line represents the strength of the cooperation relationship between the two institutions.

As shown in **Figure 4A**, the institutions with the most publications in inflammation-related research within this field of BC were the University of Texas and the University of Iowa. With the color gradient of **Figure 4A**, we can classify organizations into different categories. Some institutions, such as Kyoto University and the University of Wisconsin, were given a darker color with a larger average appearing year (AAY). Furthermore, Sun Yat-sen University and the University of Jordan were given a lighter color with smaller AAY values. However, cooperation between different institutions seems limited to their own countries, as there appears to be little previous cross-country collaboration between various institutions. The University of Texas was the center of the institutional collaboration network map, and only it had a centrality of more than 0.1(**Figure 4B**).

### Analysis of influential authors

**Figure 5:**
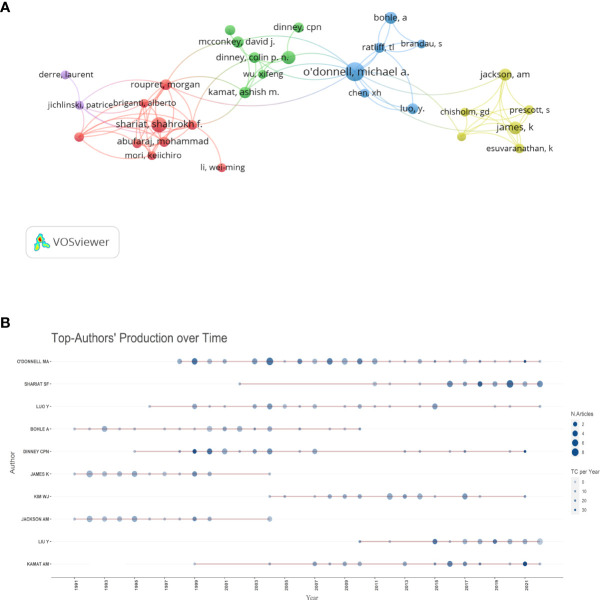
Map of the authors’ contribution. **(A)** The network visualization map of author co-citation analysis generated by VOSviewer. Each author is represented as a node, and the node size is proportional to the sum of citations. Links between two nodes indicate co-citation relationships **(B)**. The publications of the Top10 authors in the field over the years. The size of the circle indicates the number of documents (N. documents) and the color shading indicates the total number of citations (TC).

As **Table 2** shows, O’Donnell, Michael A. was the most prolific author, but this author wasn’t among the top 10 co-cited authors. Despite its small number of publications, Lamm, Donald remained the most co-cited author. Furthermore, 7 of the top 10 authors in terms of the number of publications and 6 of the top 10 co-cited authors came from the USA. The authors’ co-citation relationships are shown in **Figure 5A**, and the top 3 authors in terms of total link strength are Lamm, Donald, Bohle, Andreas, and Herr, Harry. **Figure 5B** shows that O’Donnell, Michael A. published many papers from 2000 to 2022, Jackson, Annette M. published documents mainly before 2004, and Liu, Youhua, Shariat, and Shahrokh F have published numerous articles in recent years.

**Table 2:** The top10 authors and co-cited authors of inflammation-related research within the field of bladder cancer.

RANK	AUTHOR	COUNT	H-INDEX	RANK	CO-CIETED AUTHOR	CO-CITATION	H-INDEX
1	O’Donnell, Michael A(USA)	60	45	1	Lamm, Donald(USA)	846	52
2	Shariat, Shahrokh F.(USA)	37	105	2	Herr, Harry(USA)	541	72
3	Luo, Yi(USA)	30	13	3	Morales, A(USA)	501	39
4	Bohle, A. (USA)	26	39	4	Bohle, Andreas(GERMANY)	498	40
5	Dinney, Colin P.(USA)	24	66	5	Ratliff, Therese L.(USA)	491	48
6	James, K(UK)	24	29	6	Sylvester, Richard(BELGIUM)	471	69
7	Kim, Wun-Jae(South Korea)	23	47	7	Babjuk, Marko(AUSTRIA)	445	38
8	Jackson, Annette M.(USA)	23	27	8	Jackson, Annette M.(USA)	436	27
9	Liu, Youhua (China)	23	12	9	Witjes, Fred(Netherlands)	425	94
10	Kamat, Ashish M.(USA)	22	57	10	Bellmunt, Joaquim(USA)	275	81

The table is sorted by the number of publications. The number of citations was only counted for 4301 publications from the included local databases.

### Most active journals

**Figure 6:**
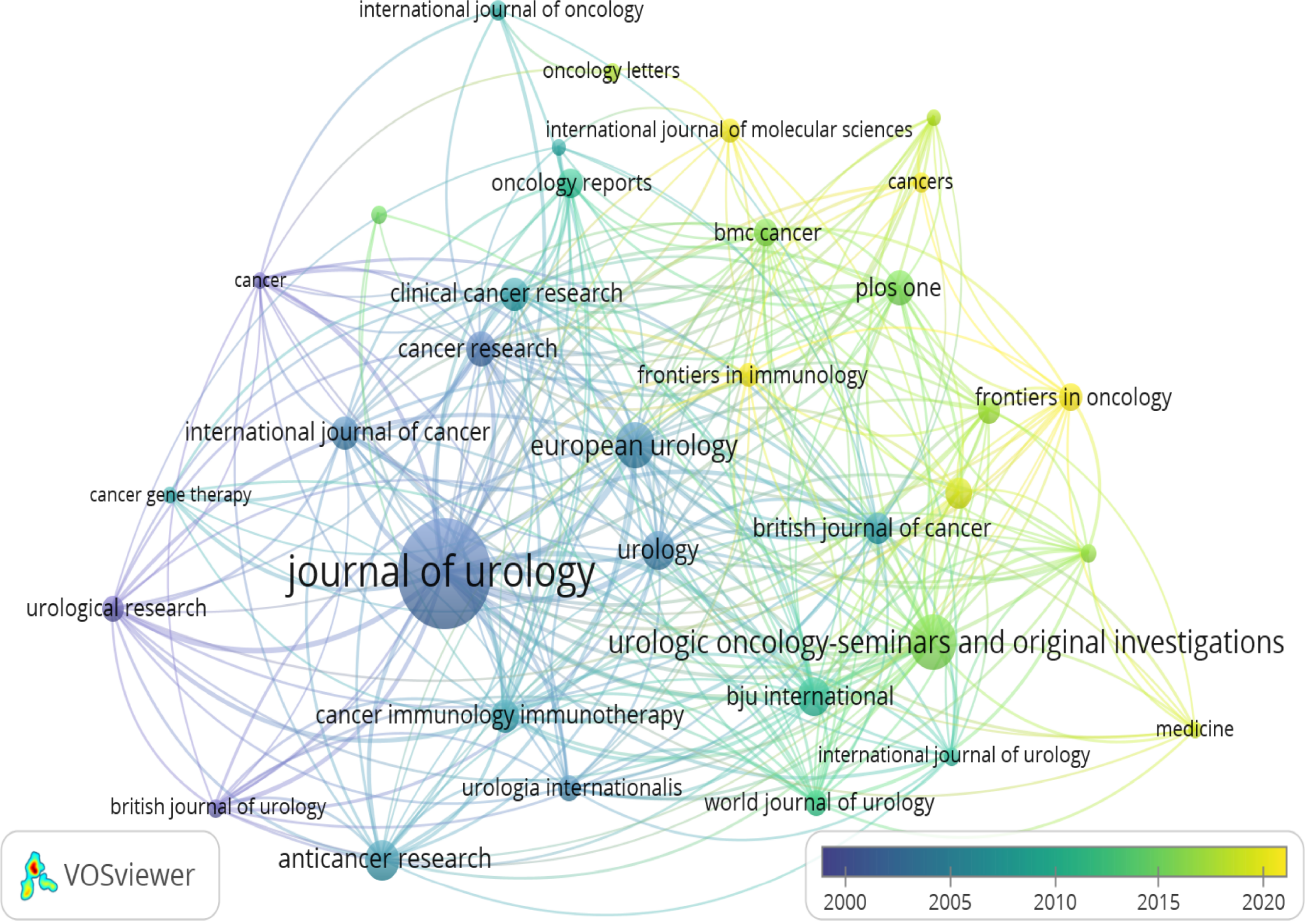
Network visualization map of journal co-occurrence. Network visualization map of journal co-occurrence analysis generated by VOSviewer. Each node represents a journal, and the larger size of the node represents the larger number of publications in that journal. The density of links between two nodes reflects the co-authorship relationship between journals.

**Figure 7:**
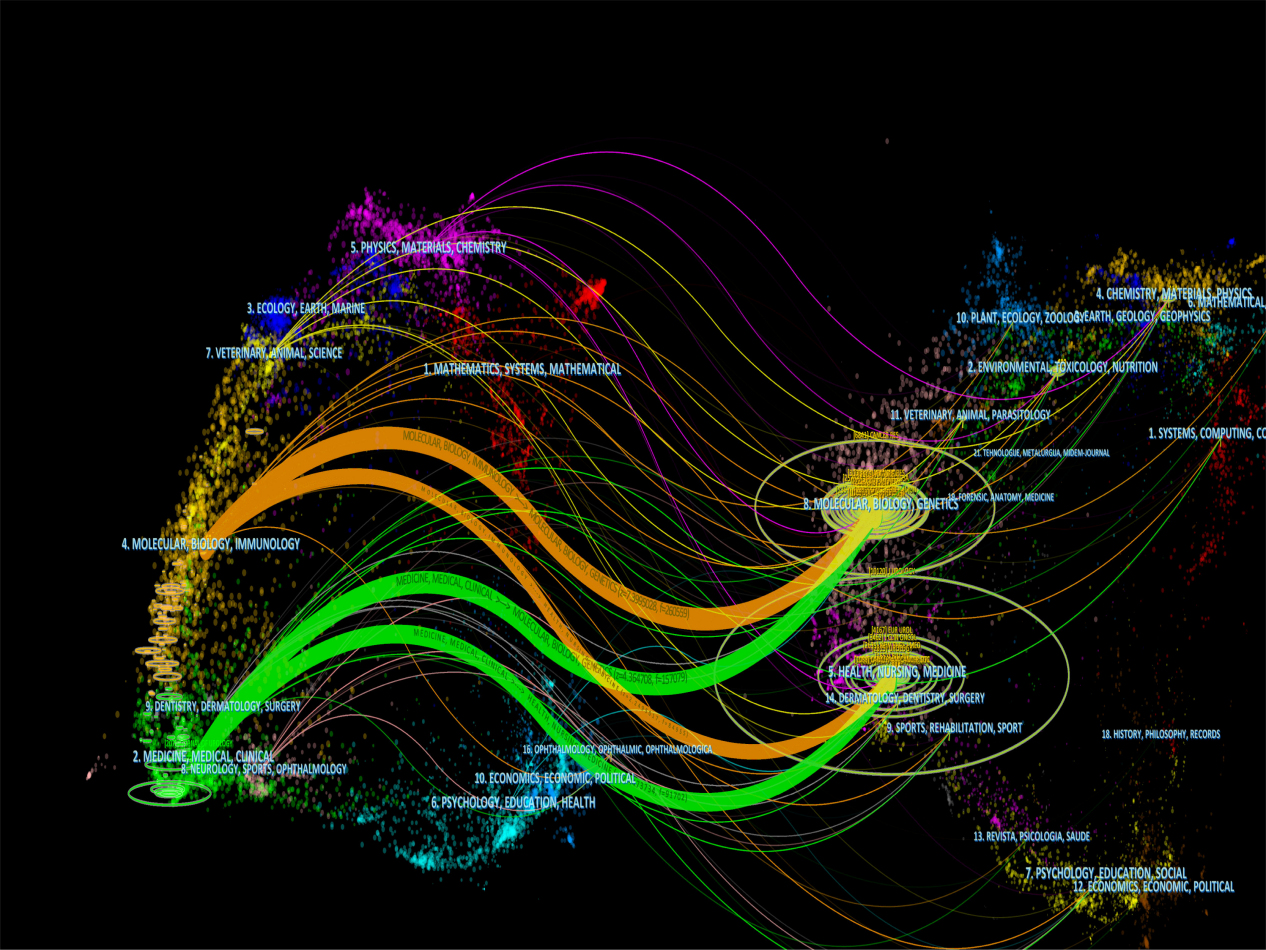
The dual-map overlay of journals. The dual-map overlay of journals related to inflammation-related research within the field of bladder cancer. Notes: The citing journals were on the left, and the cited journals were on the right.

From **Table 3**, we can see the basic information about the top 10 most published journals and the top 10 most cited journals within the field of inflammation and BC. The *Journal of urology* was the field’s most published and cited. More than 7 of the top 10 most influential journals were categorized in the Q1 or Q2 journal citation reports (JCR) region. In addition, **Figure 6** shows the co-occurrence network of the journals. As shown, *Journal of Urology*, *Urologic Oncology Seminars and Original Investigations*, and *European Urology* are the journals with the highest number of publications and are at the center of the overall co-occurrence network. **Figure 7** has four main reference paths in orange and green. This means that studies published in clinical medicine/biology/molecular journals within the field of inflammation and BC were mainly cited by papers published in genetics/healthcare/nursing journals.

**Table 3:** The top10 most productive and co-cited journals of inflammation-related research within the field of bladder cancer.

RANK	JOURNAL	N(%)	IF(2021)	JCR DICITION	RANK	CO-CIETED JOURNAL	N(%)	IF(2021)	JCR DICITION
1	Journal of Urology	256(5.95%)	7.641	Q1	1	Journal of Urology	11041(9.86%)	7.641	Q1
2	Urologic Oncology Seminars and Original Investigations	121(2.81%)	2.954	Q3	2	Cancer Research	5907(5.28%)	13.312	Q1
3	European Urology	91(2.12%)	24.344	Q1	3	European Urology	5012(4.48%)	24.344	Q1
4	Anticancer Research	79(1.84%)	2.435	Q4	4	Journal of Clinical Oncology	3672(3.28%)	50.739	Q1
5	Urology	72(1.67%)	2.633	Q3	5	Journal of Immunology	3510(3.14%)	5.43	Q2
6	BJU International	71(1.65%)	2.969	Q1	6	Clinical Cancer Research	2723(2.43%)	13.801	Q1
7	Cancer Research	67(1.56%)	13.312	Q1	7	Nature	2621(2.34%)	69.504	Q1
8	Plos One	65(1.53%)	3.752	Q2	8	New England Journal of Medicine	2612(2.33%)	176.082	Q1
9	Cancer Immunology Immunotherapy	61(1.42%)	6.633	Q1/Q2	9	Proceedings of The National Academy of Sciences of The United States of America	2548(2.27%)	12.779	Q1
10	Clinical Cancer Research	60(1.39%)	13.801	Q1	10	International Journal of Cancer	2490(2.22%)	7.316	Q1

The table is sorted by the number of publications. The number of citations was only counted for 4301 publications from the included local databases. Percentages are of the number of publications and citations. Impact factors and JCR partitions of journals were obtained from the Journal Citation Reports 2021 (JCR, http://clarivate.com/Products/WebofScience).

**Figure 8:**
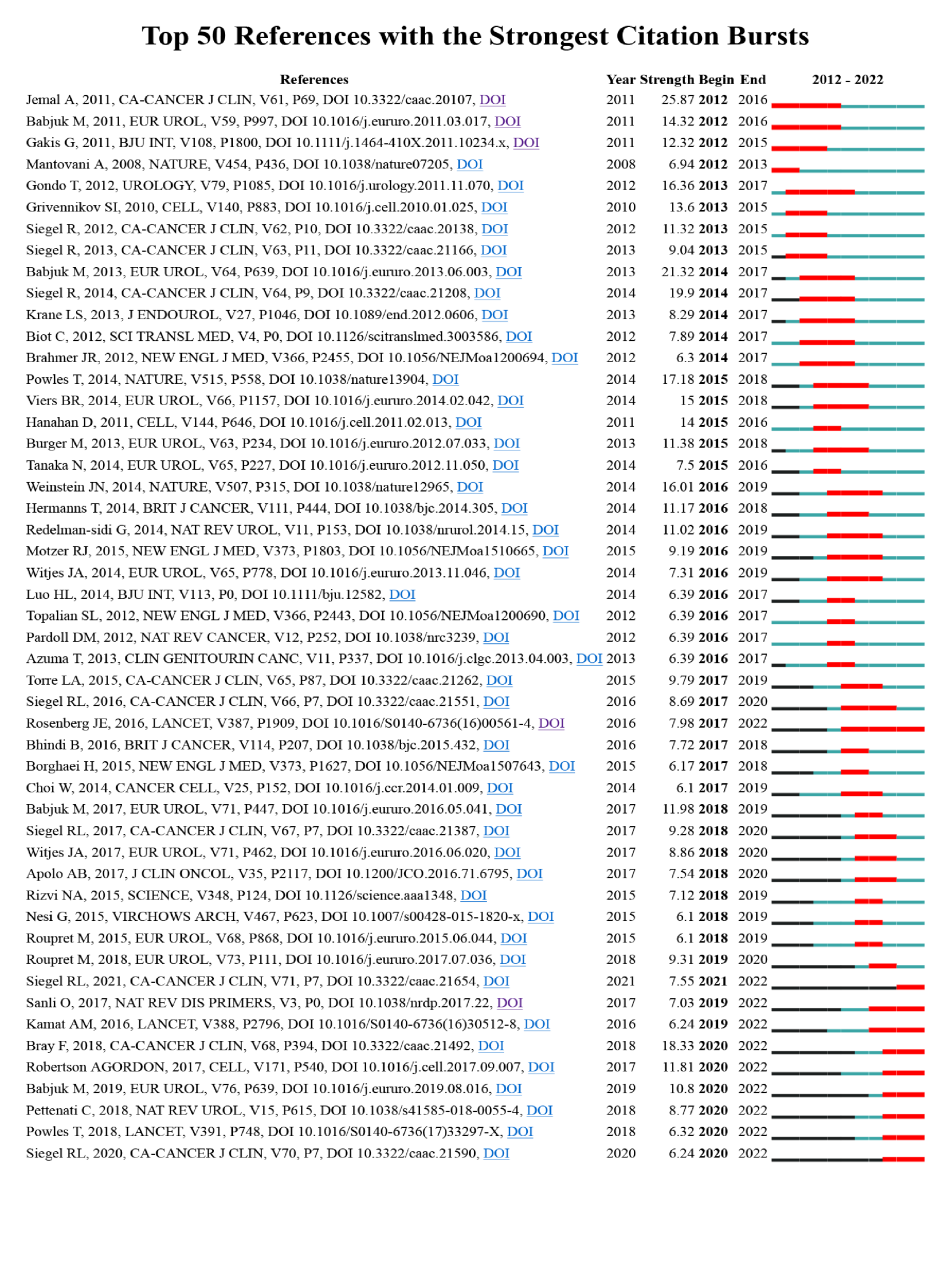
Top 50 references with the strongest citation bursts in the last decade. The dual-map overlay of journals. The red bars in this figure represent the burst period of the reference.

References with the strongest citation bursts **Table 4** shows the top 10 most cited papers in the inflammation and BC field. Only papers with more than 200 citations were included, and only one was cited more than 500 times. Most of the articles in the table were published before 2010. Additionally, we conducted a citation burst analysis using CiteSpace to find the Top 50 references with the strongest citation bursts in the last decade. (**Figure 8**). Notably, the article published by Jemal A in 2011 was the most cited literature about inflammation and BC, with the highest citation intensity in 10 years. This article received much attention mainly from 2012 to 2016 (34). Likewise, Robertson AG’s paper on the inflammation-related molecular characterization of muscle-invasive BC has been heavily cited since 2020, and this article is a recent hot topic in the field (35).

**Table 4:** Top 10 references with the strongest citation for inflammation-related research within the field of bladder cancer.

RANK	TITILE	AUTHOR	JOUNAL	CITATIONS
1	Incidence and treatment of complications of bacillus-calmette-guerin intravesical therapy in superfical bladder cancer (24)	Lamm, Donald	JOURNAL OF UROLOGY	585
2	The mechanism of action of BCG therapy for bladder cancer-a current perspective (25)	Redelman-Sidi Gil	NATURE REVIEWS UROLOGY	401
3	BCG immunotherapy of bladder cancer: 20 years on (26)	Alexandroff, Anton	LANCET	376
4	Interferon-alpha-mediated down-regulation of angiogenesis-related genes and therapy of bladder cancer are dependent on optimization of biological dose and schedule (27)	Slaton, Joel	CLINICAL CANCER RESEARCH	301
5	Relationship between schistosomiasis and bladder cancer (28)	Mostafa, Mohamed H.	CLINICAL MICROBIOLOGY REVIEWS	299
6	Regression of bladder tumors in mice treated with interleukin-2 gene modified tumor cells (29)	CONNOR, J	JOURNAL OF EXPERIMENTAL MEDICINE	276
7	Celecoxib inhibits N-butyl-N-(4-hydroxybutyl)-nitrosamine-induced urinary bladder cancer in male B6D2F1 mice and female Fischer-344 rats (30)	Grubbs, Clinton J.	CANCER RESEARCH	265
8	Immune mechanisms in bacillus Calmette-Guerin immunotherapy for superficial bladder cancer (31)	Bohle, Andreas	JOURNAL OF UROLOGY	245
9	Molecular Drivers of the Non-T-cell-Inflamed Tumor Microenvironment in Urothelial bladder cancer (32)	Sweis, Randy F.	CACNER IMMUNOLRES	225
10	Paclitaxel enhances the effects of the anti-epidermal growth factor receptor monoclonal antibody ImClone C225 in mice with metastatic human bladder transitional cell carcinoma (33)	Inoue K	CLINICAL CANCER RESEARCH	218

The table is sorted by the number of citations. The number of citations was only counted for 4301 publications from the included local databases.

### Analysis of co-occurring keywords

**Figure 9:**
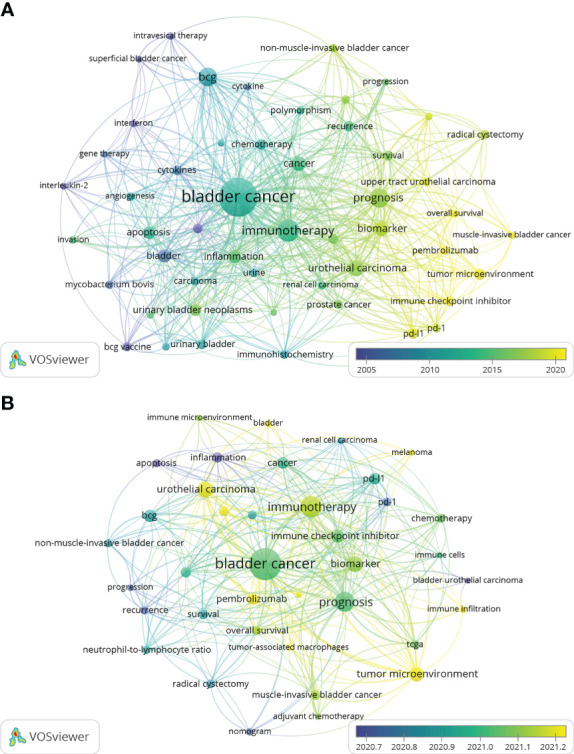
Map of the keywords ‘ contribution. **(A)** Overlay visualization map of keywords analysis in inflammation-related research within the field of bladder cancer based on the VOSviewer. The node size is proportional to the sum of occurrence times. The color of each node implies the average appearing year according to the AAY. **(B)** Overlay visualization map of keywords analysis in inflammation-related research within the field of bladder cancer in the last 3 years.

**Figure 10:**
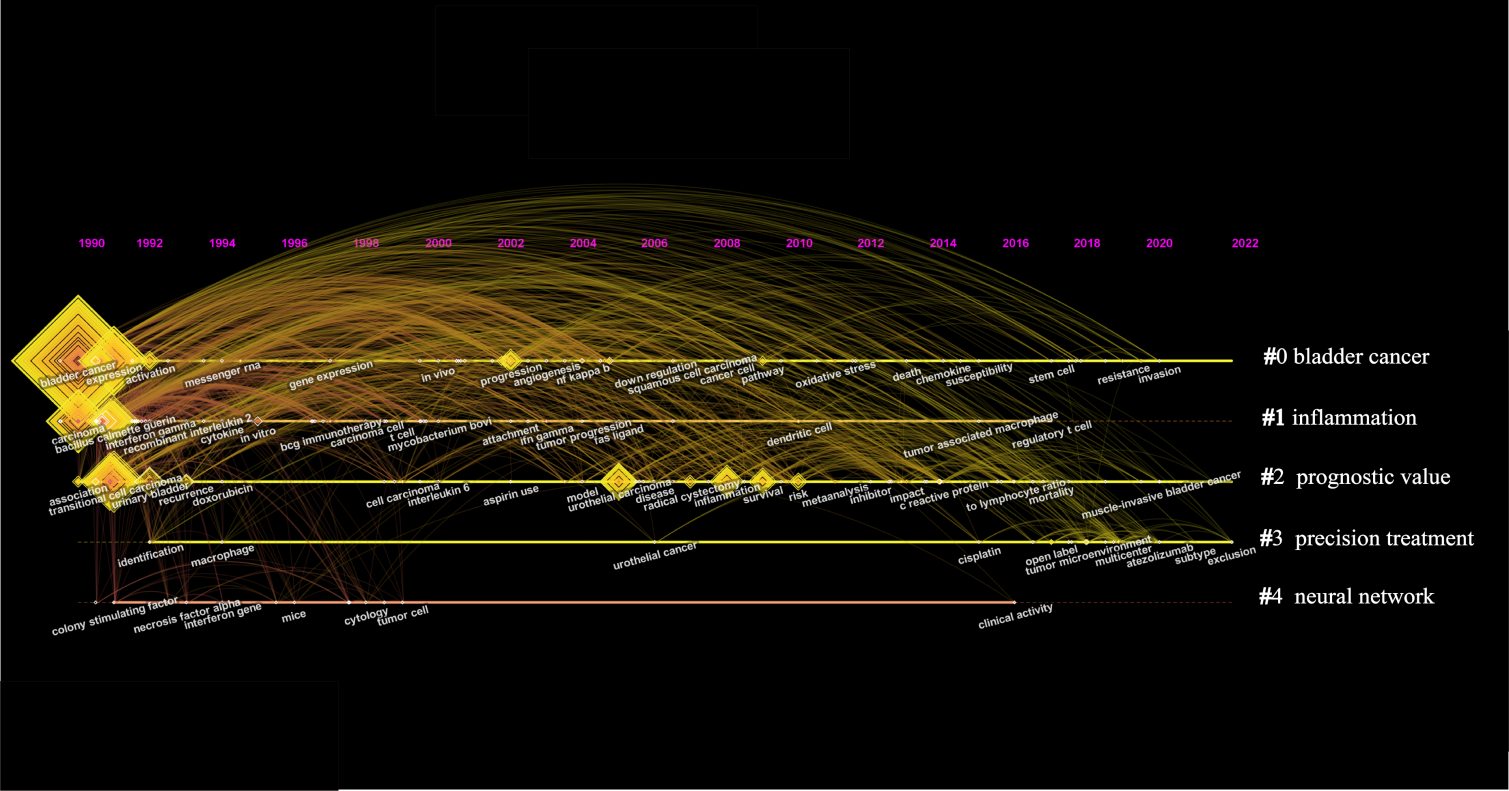
The timeline view map of keywords co-citation. CiteSpace generated the map. The years from 1979 to 2022 are arranged horizontally at the top. Each point represents an author keyword, and connecting lines represent the presence of co-linear relationships. This view map implies the differences in the appearance time point of 5 clusters—the bigger the circle size, the more research on the topic. The LLR algorithm derives the clusters.

The top 3 most frequent keywords in inflammation-related research within the field of bladder cancer are “Bladder Cancer”, “BCG”, and “Immunotherapy”, ”Immune Checkpoint Inhibitor, ” “Tumor Microenvironment,”and”Biomarker” were the top 10 keywords in the average publication year of keyword occurrence(**Table 5**). 73author keywords with at least 20 occurrences were extracted and visualized by VOSviewer, and this map reflects the hot spots at different times (**Figure 9A**). Importantly, this map shows the transition of keywords from older “interleukin 2”, “BCG”, and “TNFα” to the newer “pdl-1”, “tumor microenvironment”, and “immune checkpoint inhibitor”. **Figure 9B** shows the keyword co-occurrence network in this field in the last 3 years, and it can be seen that “tumor microenvironment” and “immune checkpoint inhibitors” are still relatively new keywords. As shown in **Figure 10**, Keywords were divided by citespace into 5 clusters based on relevance, the first cluster was “#0 bladder cancer,” followed by “#1 inflammation” and “#2 prognostic value.” The year in which the node representing the keyword appears is the first year in which it appears in the database of this study, the shifts between nodes reveal the shifting focus in inflammation-related research within the field of BC. From 1992 to 2002, research in inflammation and BC focused on topics related to “BCG” (36), “interleukins” (37), and “gene expression” (38). From 2002 to 2012, “prognosis” (39), “biomarker” (40), and “survival” received more attention. In addition, “tumor microenvironment” (41), “ muscle-invasive bladder cancer” (42), and “invasion” (43) became new foci in the last decade, from 2012-2022.Additionally, **Supplementary Figure 1** shows the relationship between authors, institutions, and keywords in inflammation-related research within the field of BC.O’Donnell, Michael A., Dinney, Colin P. and Kamat, Ashish M. from the University of Texas and the University of Iowa focused on the keywords including “expression”, “survival”, “biomarkers”, “immunotherapy”, and “BCG”.

**Table 5:** The top 10 terms with the most frequently occurring and newest average publication year of inflammation-related research within the field of bladder cancer.

RANK	TERM	COUNT	RANK	TERM	Average publication year
1	Bladder Cancer	1306(12.21%)	1	Immune Checkpoint Inhibitor	2020.02
2	BCG	385(3.60%)	2	Pembrolizumab	2020.09
3	Immunotherapy	285(2.66%)	3	Muscle-invasive Bladder Cancer	2020.01
4	Cancer	259(2.42%)	4	Tumor Microenvironment	2019.05
5	Bladder	191(1.79%)	5	Overall Survival	2019.08
6	Prognosis	162(1.51%)	6	PD-L1	2019.05
7	Inflammation	152(1.42%)	7	Upper Tract Urothelial Carcinoma	2018.04
8	Urothelial Carcinoma	129(1.21%)	8	Neutrophil-to-Lymphocyte ratio	2018.04
9	Biomarker	124(1.16%)	9	Non-Muscle-Invasive Bladder Cancer	2018.01
10	Apoptosis	107(1.01%)	10	Biomarker	2017.08

The data in the table are from VOSviewer. The percentage is the percentage of all keywords appearing in 4301 publications.

## Discussion

### Global publication trends in inflammation-related research within the field of BC

In bibliometrics, changes in the number of publications can reflect the development of a field (16). From 1972 to 1990, which we call the nascent period, 95 articles were published. At this time, the number of publications is volatile, with an average of only 5.5 publications per year. This may herald the initial emergence of research related to inflammation and BC during this period. From 1991 until 2011, we refer to this period as a stable upward period. During this time, the number of publications steadily increased to an average of 85 per year. The third phase is the period of rapid growth from 2012 to the present day. During this period, inflammation-related research within the field of BC attracted the attention of many scientists, and the average number of publications in this field exceeded 100 per year. In addition, we re-searched the relevant papers published in 2022 according to our search strategy and manually screened them. We found 380 articles and reviews published in the field of inflammation and BC in 2022, which is not much different from the prediction result of our fitting curve.This indicates that our fitted curves have some predictive function.

### Major contributors in inflammation-related research within the field of BC

#### Countries and institutions

Almost all of the top 20 countries in **Table 1** are developed countries. They have made an outstanding contribution to inflammation-related research within the field of BC. The USA had the highest number of published literature and citations and had the most significant link strength in the map of country co-authorship. From **Figure 3**, we can see that in 2015, China started to publish more articles per year than the USA (**Figure 3A**). However, the average number of citations per document in China was only 20.8 (**Table 1**). This evidence demonstrates that the United States is the most significant contributor to the field, followed by China. This was also consistent with the findings of many other bibliometric studies (44, 45).

As shown in **Figures 4A, B**, the University of Texas is at the center of the network of institutional collaboration with betweenness centrality> 1, and it has the largest number of publications. Therefore, these statements indicate that the University of Texas is the foremost research institution in inflammation and BC. However, the intensity of cooperation between institutions from different countries still needs to be increased. We hope that countries can strengthen global collaborations to promote the prosperity of research in this field.

#### Authors

**Table 2** shows that O’Donnell, Michael A is the author with the highest number of publications. Importantly, he published many articles in this field from 1999 to 2022 (**Figure 5A**). His paper BCG immunotherapy of bladder cancer: 20 years on published in *LANCET*, summarizes the progress of bacillus Calmette-Guerin (BCG) treatment of bladder cancer from 1979 to 1999, making a massive contribution to the field of inflammation and BC (26).However, his other papers published in the field are rarely cited.Although having many publications, he is not among his field’s top 10 co-cited authors. Lamm, Donald is the top 1 co-cited author in the field, although he does not have as many publications and still occupies core locations in the co-citation network (**Figure 5A**). His publication on BCG treatment of BC complications through inflammatory response was the most cited article in this field,which describes the inflammatory mechanisms of BCG for BC and briefly analyzes the complications of immunotherapy for BC (24). In summary, the influence of an author may not be solely determined by the number of publications; O’Donnell, Michael A and Lamm, Donald are all reputable authors within the field of inflammation and BC.

#### Journals

The *Journal of Urology* is the most published and cited journal in the field of inflammation and BC and is also the most active journal (**Table 3**). It is worth mentioning that in another bibliometric study on urology, the *Journal of urology* was also the most influential (46). More than 7 of the top 10 most influential journals were categorized as Q1 or Q2 journals. This finding indicates the relatively high level of investigations receiving attention in inflammation-related research within this field of BC. In addition, the *Journal of Urology*, *Cancer Research*, and *Journal of Clinical Oncology* was the Top 3 journals with the co-citation intensity, this may be related to the fact that they were categorized as Q1 and have a high IFs. Therefore, this may indicate that the articles published in these journals deserve more attention.

### Hotspot evolution, knowledge structure, and emerging topics

#### References and keywords analysis

Reference analysis and keyword analysis are among the important methods of bibliometric research by which we can analyze the evolution of hot spots within our field of study (47). Six of the ten most cited articles in the field of inflammation and BC were related to immunotherapy, while two were related to the tumor microenvironment (**Table 4**). As seen in **Figure 8**, immunotherapy continues to see an explosion of high-quality articles published or cited in recent years. Similarly, studies on inflammation-related biomarkers and the tumor microenvironment have received much attention in the last 10 years. In the keyword analysis, we can see from **Table 5** that immunotherapy is the 3th-ranked keyword. In contrast, BCG, within the scope of immunotherapy, was the 2nd-ranked keyword, and biomarkers was in the 9th keywords. In **Figure 9A**, we found that immunotherapy and biomarkers whose related keywords are located in the figure’s center are the keyword network’s core. At the same time, tumor markers are colored yellow, representing a later year of appearance. **Figure 10** illustrates the changing hotspots within the field of inflammation and BC, and it is clear to see that immunotherapy has been a hotspot of interest for the past few decades. Notably, inflammation-related biomarkers emerged as a hotspot more than 10 years ago, and the tumor microenvironment only started to gain attention a few years ago. In summary, immunotherapy is a topic that has been widely studied in inflammation and BC. Moreso, inflammation-related biomarkers are gradually gaining attention during the development of this field, and tumor microenvironment is an emerging hotspot within the field.

##### Immunotherapy

Since the 1970s, research related to immunotherapy within the field of inflammation and BC has been growing rapidly. The earliest immunotherapy for BC was BCG, which inhibits BC cell proliferation by directing the inflammatory response through the attachment of fibronectin and integrin α5β1 to urinary epithelial cells (25) here were also many studies on BCG in the burst citation literature in the last decade (**Figure 8**) **(** 25, 48, 49). In addition to BCG, immunotherapy commonly used for BC includes immune checkpoint inhibitors and cytokines. Immune checkpoint inhibitors, including PD-1, PD-L1, and CTLA4, inhibit the tumor’s division, growth, and invasion by affecting the immune response of the tumor (50). The study of immune checkpoint inhibitors, especially PD-1 and PD-L1, was an emerging hotspot in inflammation and BC. Many relevant papers have been published recently and are heavily cited in the last decade (50–54).Moreover, the central cytokines studied within the field of inflammation and BC are interleukins (IL), interferons (INF), and tumor necrosis factor (TNF). Among them, Slaton, Joel’s study on INF was the 5th most cited article (**Table 4**). This study demonstrated that IFN-α affects the anti-angiogenic activity of BC cells, which is likely to be a new direction for BC treatment (27). Moreso, studies on IL have been long-standing. As shown in **Figure 10**, Research on IL first appeared in 1889 and continued to attract scholarly interest until 2022 (55–57). Therefore, immunotherapy has been the focus of attention in inflammation and BC and deserves the focus of scientists.

##### Inflammation related biomarkers

Biomarkers are biochemical indicators that can reflect specific structural or functional changes in the body (58). Notably, there is a desire to find a perfect biomarker to predict BC’s occurrence, progression, and prognosis (59). The neutrophil-to-lymphocyte ratio (NLR) is one of the most exciting inflammatory biomarkers, and high NLR decreases lymphocyte responsiveness to malignancy, thus affecting prognosis (60). **Figure 9A** shows that NLR has received significant attention in the last two years. In addition, many of the 50 most cited papers in the previous ten years investigated the relationship between NLR and BC prognosis (60–62) (**Figure 8**). C-reactive protein (CRP) is another inflammation-related biomarker. CRP is part of the innate immune system and has received a great deal of scholarly attention in inflammation and BC. A study by Gakis,Georgios demonstrated the prognostic impact of CRP on BC and has been heavily cited in the last decade (63). To date, no biomarkers can perfectly predict BC’s diagnosis, treatment and prognosis. Therefore we require added studies to identify better and more comprehensive markers.

##### Tumor microenvironment

The microenvironment of BC is a complex of multiple factors that promote and inhibit anti-tumor immune responses, interacting through inflammation-related immune checkpoint molecules, cytokines, and chemokines (64). The tumor microenvironment has received increasing attention in inflammation and BC research in recent years (65–67). The 10th most cited article in this study demonstrated (**Table 4**) that BC’s tumor microenvironment changes are associated with inflammatory cells (including regulatory T cells, tumor-associated macrophages, and other immunosuppressive cells). This finding has also been widely recognized by peers (32). In addition, good progress has recently been made regarding studying giant tumor cells within the tumor microenvironment. Additionally, massive infiltration of M2 phenotype tumor giant cells in BC has been demonstrated to be associated with tumor aggressiveness (68). These results suggest that the tumor microenvironment may be a new direction for a future breakthrough in inflammation and BC.

### Future research directions and hotspots

One of the standard methods of bibliometric research is to analyze the keywords of past published literature to derive future research directions and hotspots (14).In this study, we found that “tumor microenvironment”, “immune checkpoint inhibitors”, and “biomarker” were the keywords that were mentioned more often and appeared in later years (**Figure 9A**).We performed keyword co-occurrence in **Figure 9B** for the literature published in the last 3 years, and we found that “tumor microenvironment,” “immune checkpoint inhibitors,” and “biomarker” were still the keywords that appeared many times.As shown in **Table 5**, “tumor microenvironment,” “immune checkpoint inhibitors,” and “biomarker” average publication year of publication ranked 1st, 4th, and 10th among all keywords. In the field of inflammation and BC, biomarkers are primarily associated with inflammation, and immune checkpoint inhibitors are a type of immunotherapy. By analyzing the papers published in the past, we found that tumor microenvironment, immunotherapy, and inflammation-related biomarkers were the hotspots of research in the past years. Therefore, it is reasonable to assume that they also agree to represent the future research hotspots and development directions. In a recently published study on immunotherapy for bladder cancer, the potential of the tumor microenvironment as a future research focus was also highlighted, which aligns with our own findings (18).It suggests that our study is significant and could provide valuable insights for clinicians and researchers in the field of inflammation and BC. In the future, significant progress is expected to be made by researchers in the three areas of tumor microenvironment, immunotherapy, and inflammation-related biomarkers.

### Strengths and limitation

This study is the first to describe and visualize the knowledge landscape about inflammation-related research within the field of BC. Compared to previous reviews and meta-analyses, our study has more advantages. We used several bibliometric tools to visualize the data, adding specificity and richness to the results. As with other studies, this study has some noted limitations. Firstly, our data were obtained from the WOS database, so we may need to include other relevant literature. Secondly, the language of publications included in this study is limited to English, which may lead us to overlook some essential publications. Finally, there was no uniform standard for manual selection, which can influence selection bias when obtaining the data.

## Conclusions

This study clarifies the contribution of countries, institutions, authors, and journals in the field of inflammation and BC through a bibliometric approach. It also identifies research hotspots and frontiers in the field. Notably, research topics such as “immunotherapy”, “biomarkers”, and “tumor microenvironment” are hotspots and frontiers in the field of inflammation and BC. Therefore, these findings can help researchers to understand more clearly the relationship between inflammation and BC.

## Data availability statement

The raw data supporting the conclusions of this article will be made available by the authors, without undue reservation.

## Author contributions

ZD and NT (First Author): conceptualization, methodology, software, investigation, formal analysis, writing - original draft. WX: data curation, writing - original draft. Xu Lei: visualization, investigation. TZ: software, validation. NY (Corresponding Author):conceptualization, funding acquisition, resources, supervision, writing - review & editing. ZD and NT have contributed equally to this work and share first authorship. All authors contributed to the article and approved the submitted version.

## References

[1] Sanli O Dobruch J Knowles MA Burger M Alemozaffar M Nielsen ME. Bladder cancer. Nat Rev Dis Primers (2017) 3:19. doi: 10.1038/nrdp.2017.22 28406148

[2] Sung H Ferlay J Siegel RL Laversanne M Soerjomataram I Jemal A. Global cancer statistics 2020: globocan estimates of incidence and mortality worldwide for 36 cancers in 185 countries. Ca-a Cancer J Clin (2021) 71(3):209–49. doi: 10.3322/caac.21660 33538338

[3] Kwan ML Hague R Young-Wolff KC Lee VS Roh JM Ergas IJ. Smoking behaviors and prognosis in patients with non-Muscle-Invasive bladder cancer in the be-well study. JAMA Netw Open (2022) 5(11):15. doi: 10.1001/jamanetworkopen.2022.44430 PMC9713602 36449286

[4] Baris D Karagas MR Verrill C Johnson A Andrew AS Marsit CJ. A case-control study of smoking and bladder cancer risk: emergent patterns over time. JNCI-J Natl Cancer Inst (2009) 101(22):1553–61. doi: 10.1093/jnci/djp361 PMC2778671 19917915

[5] Leibovici D Grossman HB Dinney CP Millikan RE Lerner S Wang YF. Polymorphisms in inflammation genes and bladder cancer: from initiation to recurrence, progression, and survival. J Clin Oncol (2005) 23(24):5746–56. doi: 10.1200/jco.2005.01.598 16110031

[6] Grivennikov SI Greten FR Karin M. Immunity, inflammation, and cancer. Cell (2010) 140(6):883–99. doi: 10.1016/j.cell.2010.01.025 PMC2866629 20303878

[7] Suttmann H Riemensberger J Bentien G Schmaltz D Stockle M Jocham D. Neutrophil granulocytes are required for effective bacillus calmette-guerin immunotherapy of bladder cancer and orchestrate local immune responses. Cancer Res (2006) 66(16):8250–7. doi: 10.1158/0008-5472.Can-06-1416 16912205

[8] Huang XX Pan T Yan LL Jin T Zhang RN Chen B. The inflammatory microenvironment and the urinary microbiome in the initiation and progression of bladder cancer. Genes Dis (2021) 8(6):781–97. doi: 10.1016/j.gendis.2020.10.002 PMC8427242 34522708

[9] Sui XB Lei LM Chen LX Xie T Li X. Inflammatory microenvironment in the initiation and progression of bladder cancer. Oncotarget (2017) 8(54):93279–94. doi: 10.18632/oncotarget.21565 PMC5696263 29190997

[10] Wigner P Grebowski R Bijak M Saluk-Bijak J Szemraj J. The interplay between oxidative stress, inflammation and angiogenesis in bladder cancer development. Int J Mol Sci (2021) 22(9). doi: 10.3390/ijms22094483 PMC8123426 33923108

[11] Zhixuan D Ning Y. Research progress on the relationship between inflammatory markers and prognosis of bladder cancer. Med Sci J Cent South China (2022) 50(6):621–4. doi: 10.15972/j.cnki.43-1509/r.2022.04.040

[12] Huang XL Tao YT Gao JM Zhou XG Tang SM Deng CW. Ubc9 coordinates inflammation affecting development of bladder cancer. Sci Rep (2020) 10(1). doi: 10.1038/s41598-020-77623-9 PMC7691338 33244139

[13] Zhang WT Wang RL Ma WC Wu Y Maskey N Guo YD. Systemic immune-inflammation index predicts prognosis of bladder cancer patients after radical cystectomy. Ann Trans Med (2019) 7(18). doi: 10.21037/atm.2019.09.02 PMC6803204 31700867

[14] Donthu N Kumar S Mukherjee D Pandey N Lim WM. How to conduct a bibliometric analysis: an overview and guidelines. J Bus Res (2021) 133:285–96. doi: 10.1016/j.jbusres.2021.04.070

[15] Sugimoto CR Ahn YY Smith E Macaluso B Lariviere V. Factors affecting sex-related reporting in medical research: a cross-disciplinary bibliometric analysis. Lancet (2019) 393(10171):550–9. doi: 10.1016/s0140-6736(18)32995-7 30739690

[16] Mainwaring A Bullock N Ellul T Hughes O Featherstone J. The top 100 most cited manuscripts in bladder cancer: a bibliometric analysis (Review article). Int J Surg (2020) 75:130–8. doi: 10.1016/j.ijsu.2020.01.128 31991242

[17] Lee YL Chien TW Wang JC. Using sankey diagrams to explore the trend of article citations in the field of bladder cancer: research achievements in China higher than those in the united states. Med (Baltimore) (2022) 101(34):9. doi: 10.1097/md.0000000000030217 PMC9410696 36042603

[18] Qiu QQ Deng C Li HQ Qiu JH Shen ZF Ding YQ. The global research of bladder cancer immunotherapy from 2012 to 2021: a bibliometric analysis. Front Oncol (2022) 12:999203. doi: 10.3389/fonc.2022.999203 36452503 PMC9703076

[19] Shen ZF Hu JT Wu HY Chen ZS Wu WX Lin JY. Global research trends and foci of artificial intelligence-based tumor pathology: a scientometric study. J Transl Med (2022) 20(1):17. doi: 10.1186/s12967-022-03615-0 36068536 PMC9450455

[20] Zhong WB Shen ZF Wu YX Mao XM Kong JQ Wu WX. Knowledge mapping and current trends of immunotherapy for prostate cancer: a bibliometric study. Front Immunol (2022) 13:1014981. doi: 10.3389/fimmu.2022.1014981 36389756 PMC9647028

[21] Wang L Zhou S Liu Y Li Y Sun X. Bibliometric analysis of the inflammatory mechanism in aortic disease. Rev Cardiovasc Med (2022) 23(2):67. doi: 10.31083/j.rcm2302067 35229558

[22] Xu H Ju J Zhang J Wang X Zhang T Tian W. Research landscape on atherosclerotic cardiovascular disease and inflammation: a bibliometric and visualized study. Rev Cardiovasc Med (2022) 23(9). doi: 10.31083/j.rcm2309317 PMC11262408 39077721

[23] Lim M Carollo A Dimitriou D Esposito G. Recent developments in autism genetic research: a scientometric review from 2018 to 2022. Genes (2022) 13(9):22. doi: 10.3390/genes13091646 PMC9498399 36140813

[24] Lamm DL Vandermeijden APM Morales A Brosman SA Catalona WJ Herr HW. Incidence and treatment of complications of bacillus-Calmette-Guerin intravesical therapy in superficial bladder-cancer. J Urol (1992) 147(3):596–600. doi: 10.1016/s0022-5347(17)37316-0 1538436

[25] Redelman-Sidi G Glickman MS Bochner BH. The mechanism of action of bcg therapy for bladder cancer-a current perspective. Nat Rev Urol (2014) 11(3):153–62. doi: 10.1038/nrurol.2014.15 24492433

[26] Alexandroff AB Jackson AM O'Donnell MA James K. Bcg immunotherapy of bladder cancer: 20 years on. Lancet (1999) 353(9165):1689–94. doi: 10.1016/s0140-6736(98)07422-4 10335805

[27] Slaton JW Perrotte P Inoue K Dinney CPN Fidler IJ. Interferon-Alpha-Mediated down-regulation of angiogenesis-related genes and therapy of bladder cancer are dependent on optimization of biological dose and schedule. Clin Cancer Res (1999) 5(10):2726–34. 10537335

[28] Mostafa MH Sheweita SA O'Connor PJ. Relationship between schistosomiasis and bladder cancer. Clin Microbiol Rev (1999) 12(1):97–+. doi: 10.1128/cmr.12.1.97 PMC88908 9880476

[29] Connor J Bannerji R Saito S Heston W Fair W Gilboa E. Regression of bladder-tumors in mice treated with interleukin-2 gene-modified tumor-cells. J Exp Med (1993) 177(4):1127–34. doi: 10.1084/jem.177.4.1127 PMC2190983 8459207

[30] Grubbs CJ Lubet RA Koki AT Leahy KM Masferrer JL Steele VE. Celecoxib inhibits n-Butyl-N-(4-Hydroxybutyl)-Nitrosamine-Induced urinary bladder cancers in Male B6d2f1 mice and female Fischer-344 rats. Cancer Res (2000) 60(20):5599–602. 11059745

[31] Bohle A Brandau S. Immune mechanisms in bacillus calmette-guerin immunotherapy for superficial bladder cancer. J Urol (2003) 170(3):964–9. doi: 10.1097/01.ju.0000073852.24341.4a 12913751

[32] Sweis RF Spranger S Bao RY Paner GP Stadler WM Steinberg G. Molecular drivers of the non-T-Cell-Inflamed tumor microenvironment in urothelial bladder cancer. Cancer Immunol Res (2016) 4(7):563–8. doi: 10.1158/2326-6066.Cir-15-0274 PMC4943758 27197067

[33] Inoue K Slaton JW Perrotte P Davis DW Bruns CJ Hicklin DJ. Paclitaxel enhances the effects of the anti-epidermal growth factor receptor monoclonal antibody imclone C225 in mice with metastatic human bladder transitional cell carcinoma. Clin Cancer Res (2000) 6(12):4874–84. 11156247

[34] Jemal A Bray F Center MM Ferlay J Ward E Forman D. Global cancer statistics. Ca-a Cancer J Clin (2011) 61(2):69–90. doi: 10.3322/caac.20107 21296855

[35] Robertson AG Kim J Al-Ahmadie H Bellmunt J Guo GW Cherniack AD. Comprehensive molecular characterization of muscle-invasive bladder cancer. Cell (2017) 171(3):540. doi: 10.1016/j.cell.2017.09.007 28988769 PMC5687509

[36] Betz SA See WA Cohen MB. Granulomatous inflammation in bladder wash specimens after intravesical bacillus calmette-guerin therapy for transitional cell-carcinoma of the bladder. Am J Clin Pathol (1993) 99(3):244–8. doi: 10.1093/ajcp/99.3.244 8447285

[37] Okamoto M Kawamata H Kawai K Oyasu R. Enhancement of transformation in-vitro of a nontumorigenic rat urothelial cell-line by interleukin-6. Cancer Res (1995) 55(20):4581–5. 7553633

[38] Syrigos KN Harrington KJ Pignatelli M. Role of adhesion molecules in bladder cancer: an important part of the jigsaw. Urology (1999) 53(2):428–34. doi: 10.1016/s0090-4295(98)00527-5 9933073

[39] Offersen BV Knap MM Marcussen N Horsman MR Hamilton-Diutoit S Overgaard J. Intense inflammation in bladder carcinoma is associated with angiogenesis and indicates good prognosis. Br J Cancer (2002) 87(12):1422–30. doi: 10.1038/sj.bjc.6600615 PMC2376289 12454772

[40] Ohtake S Kawahara T Kasahara R Ito H Osaka K Hattori Y. Pretreatment neutrophil-to-Lymphocyte ratio can predict the prognosis in bladder cancer patients who receive gemcitabine and nedaplatin therapy. BioMed Res Int (2016) 2016. doi: 10.1155/2016/9846823 PMC5086366 27822480

[41] Zhu HL Jia XC Wang YP Song ZJ Wang NN Yang YL. M6a classification combined with tumor microenvironment immune characteristics analysis of bladder cancer. Front Oncol (2021) 11:714267. doi: 10.3389/fonc.2021.714267 34604051 PMC8479184

[42] Pagliarulo F Cheng PF Brugger L van Dijk N van den Heijden M Levesque MP. Molecular, immunological, and clinical features associated with lymphoid neogenesis in muscle invasive bladder cancer. Front Immunol (2022) 12:793992. doi: 10.3389/fimmu.2021.793992 35145509 PMC8821902

[43] Lin CY Lin WY Yeh SY Li L Chang CS. Infiltrating neutrophils increase bladder cancer cell invasion *Via* modulation of androgen receptor (Ar)/Mmp13 signals. Oncotarget (2015) 6(40):43081–9. doi: 10.18632/oncotarget.5638 PMC4767492 26517808

[44] Cheng KM Guo Q Yang WG Wang YL Sun ZJ Wu HY. Mapping knowledge landscapes and emerging trends of the links between bone metabolism and diabetes mellitus: a bibliometric analysis from 2000 to 2021. Front Public Health (2022) 10:918483. doi: 10.3389/fpubh.2022.918483 35719662 PMC9204186

[45] Zhang J Song LX Xu LY Fan YX Wang T Tian WD. Knowledge domain and emerging trends in ferroptosis research: a bibliometric and knowledge-map analysis. Front Oncol (2021) 11:686726. doi: 10.3389/fonc.2021.686726 34150654 PMC8209495

[46] Agarwal A Finelli R Durairajanayagam D Leisegang K Henkel R Salvio G. Comprehensive Analysis of Global Research on Human Varicocele: A Scientometric Approach. World J Mens Health (2022) 40(4):636–52. doi: 10.5534/wjmh.210202 PMC9482861 35118839

[47] Polterovich VM. Bibliometric equilibrium. Herald Russian Acad Sci (2022) 92(3):245–53. doi: 10.1134/s1019331622030194

[48] Biot C Rentsch CA Gsponer JR Birkhauser FD Jusforgues-Saklani H Lemaitre F. Preexisting bcg-specific T cells improve intravesical immunotherapy for bladder cancer. Sci Trans Med (2012) 4(137). doi: 10.1126/scitranslmed.3003586 22674550

[49] Zuiverloon TCM Nieuweboer AJM Vekony H Kirkels WJ Bangma CH Zwarthoff EC. Markers predicting response to bacillus calmette-guerin immunotherapy in high-risk bladder cancer patients: a systematic review. Eur Urol (2012) 61(1):128–45. doi: 10.1016/j.eururo.2011.09.026 22000498

[50] Massari F Di Nunno V Cubelli M Santoni M Fiorentino M Montironi R. Immune checkpoint inhibitors for metastatic bladder cancer. Cancer Treat Rev (2018) 64:11–20. doi: 10.1016/j.ctrv.2017.12.007 29407369

[51] Barone B Calogero A Scafuri L Ferro M Lucarelli G Di Zazzo E. Immune checkpoint inhibitors as a Neoadjuvant/Adjuvant treatment of muscle-invasive bladder cancer: a systematic review. Cancers (2022) 14(10). doi: 10.3390/cancers14102545 PMC9139497 35626149

[52] Chen XY Xu RS He D Zhang Y Chen HT Zhu YX. Cd8(+) T effector and immune checkpoint signatures predict prognosis and responsiveness to immunotherapy in bladder cancer. Oncogene (2021) 40(43):6223–34. doi: 10.1038/s41388-021-02019-6 34552192

[53] Brahmer JR Tykodi SS Chow LQM Hwu WJ Topalian SL Hwu P. Safety and activity of anti-Pd-L1 antibody in patients with advanced cancer. New Engl J Med (2012) 366(26):2455–65. doi: 10.1056/NEJMoa1200694 PMC3563263 22658128

[54] Pardoll DM. The blockade of immune checkpoints in cancer immunotherapy. Nat Rev Cancer (2012) 12(4):252–64. doi: 10.1038/nrc3239 PMC4856023 22437870

[55] Pizza G Severini G Menniti D Devinci C Corrado F. Tumor-regression after intralesional injection of interleukin 2 (Il-2) in bladder-cancer - preliminary-report. Int J Cancer (1984) 34(3):359–67. doi: 10.1002/ijc.2910340312 6332786

[56] Wu K Zeng J Shi XL Xie JJ Li YQ Zheng HX. Targeting tigit inhibits bladder cancer metastasis through suppressing il-32. Front Pharmacol (2022) 12:801493. doi: 10.3389/fphar.2021.801493 35069212 PMC8766971

[57] Tong SY Hu XH Li YL. Laboratory serum il-6 level is associated with clinical outcome of intravesical gemcitabine therapy in T1 non-Muscle-Invasive bladder cancer. Urol Oncol Seminars Original Invest (2022) 40(9). doi: 10.1016/j.urolonc.2022.05.020 35718638

[58] Griffiths HR Moller L Bartosz G Bast A Bertoni-Freddari C Collins A. Biomarkers. Mol Aspects Med (2002) 23(1-3):101–208. doi: 10.1016/s0098-2997(02)00017-1 12079771

[59] Bryan RT Zeegers MP James ND Wallace DMA Cheng KK. Biomarkers in bladder cancer. Bju Int (2010) 105(5):608–13. doi: 10.1111/j.1464-410X.2009.08880.x 19793380

[60] Krane LS Richards KA Kader AK Davis R Balaji KC Hemal AK. Preoperative Neutrophil/Lymphocyte ratio predicts overall survival and extravesical disease in patients undergoing radical cystectomy. J Endourol (2013) 27(8):1046–50. doi: 10.1089/end.2012.0606 23517015

[61] Azuma T Matayoshi Y Odani K Sato Y Sato Y Nagase Y. Preoperative neutrophil-lymphocyte ratio as an independent prognostic marker for patients with upper urinary tract urothelial carcinoma. Clin Genitourinary Cancer (2013) 11(3):337–41. doi: 10.1016/j.clgc.2013.04.003 23665132

[62] Gondo T Nakashima J Ohno Y Choichiro O Horiguchi Y Namiki K. Prognostic value of neutrophil-to-Lymphocyte ratio and establishment of novel preoperative risk stratification model in bladder cancer patients treated with radical cystectomy. Urology (2012) 79(5):1085–91. doi: 10.1016/j.urology.2011.11.070 22446338

[63] Gakis G Todenhofer T Renninger M Schilling D Sievert KD Schwentner C. Development of a new outcome prediction model in carcinoma invading the bladder based on preoperative serum c-reactive protein and standard pathological risk factors: the tnr-c score. Bju Int (2011) 108(11):1800–5. doi: 10.1111/j.1464-410X.2011.10234.x 21507193

[64] Hatogai K Sweis RF. The tumor microenvironment of bladder cancer. In: Birbrair A, editor. Tumor microenvironments in organs: from the brain to the skin, pt b. Advances in Experimental Medicine and Biology (2020). p. 275–90. doi: 10.1007/978-3-030-59038-3_17 PMC8345230 34185299

[65] Liu PF Cao YW Zhang SD Zhao Y Liu XG Shi HQ. A bladder cancer microenvironment simulation system based on a microfluidic Co-culture model. Oncotarget (2015) 6(35):37695–705. doi: 10.18632/oncotarget.6070 PMC4741958 26462177

[66] Paramanantham Y Chung I Said N. The role of tumour microenvironment-driven mirnas in the chemoresistance of muscle-invasive bladder cancer-a review. Urol Oncol Seminars Original Invest (2022) 40(4):133–48. doi: 10.1016/j.urolonc.2022.01.013 35246373

[67] Pan WY Han J Wei N Wu H Wang YZ Sun JN. Linc00702-mediated Dusp1 transcription in the prevention of bladder cancer progression: implications in cancer cell proliferation and tumor inflammatory microenvironment. Genomics (2022) 114(4). doi: 10.1016/j.ygeno.2022.110428 35809838

[68] Takeuchi H Tanaka M Tanaka A Tsunemi A Yamamoto H. Predominance of M2-polarized macrophages in bladder cancer affects angiogenesis, tumor grade and invasiveness. Oncol Lett (2016) 11(5):3403–8. doi: 10.3892/ol.2016.4392 PMC4841030 27123124

