# Supplementary material for: Inflammation-related research within the field of bladder cancer: a bibliometric analysis
Source: Front Oncol. 2023 Jun 7;13:1126897. doi: 10.3389/fonc.2023.1126897 (PMC10282760; doi:10.3389/fonc.2023.1126897)
Supplement: Supplementary file 1 [file DataSheet_1.pdf]

Supplementary Material

Supplementary Table 1. Search Strategy

Search Terms
TS=(inflam* OR “c reactive protein*” OR “acutephase protein*” OR interleukin* OR “tumor necrosis factor*” OR cytokine* OR interferon* OR chemokine* OR prostaglandin* OR leukotriene* OR histamine* OR neutrophil* OR macrophage* OR monocyte* OR lymphocyte*) AND TS=(“bladder cancer”or “bladder carcinoma” or“bladder tumor” or “urothelial cancer”, or “urothelial carcinoma”, or “urothelial tumor”, or “transitional cell cancer”, or “transitional cell carcinoma”or“transitional cell tumor”）

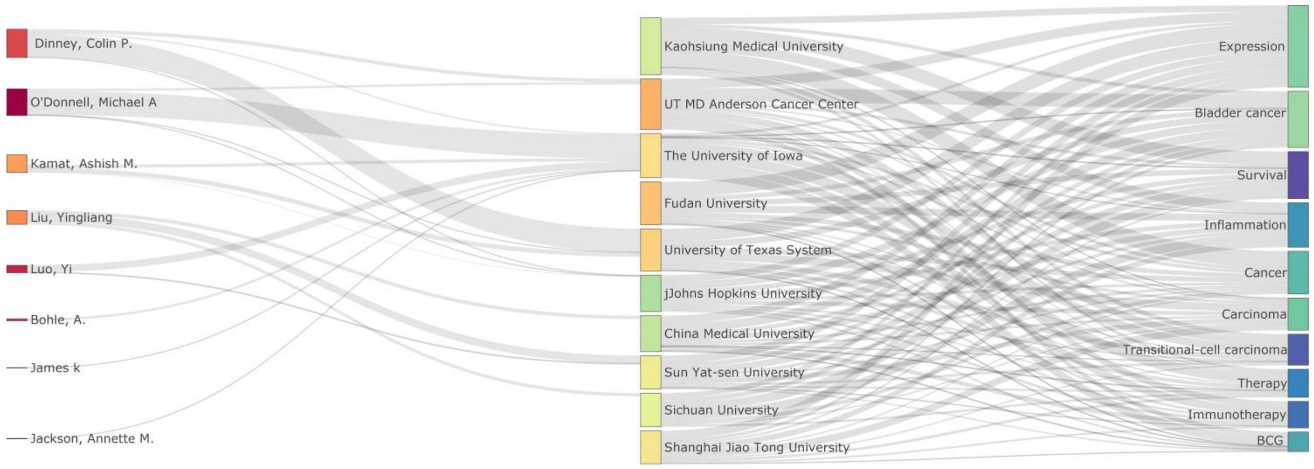

Suplementary Figure 1 .Three-field plot of the Keywords Plus analysis.

Three-field plot of the Keywords Plus analysis on inflammation-related research within the field of bladder cancer. Notes: three-field plot of the keywords plus analysis: (left field: authors; middle field: affiliations; right field: keywords plus)
